# A Theoretical and Practical Treatise on the Diseases of the Skin

**Published:** 1836-07

**Authors:** 


### Art. VI.

*A Theoretical and Practical Treatise on the Diseases of the Skin.* By P. Rayer, m.d., Physician to the Hôpital de la Charité, &c. *With an Atlas. The Second Edition, entirely remodelled.—London,* 1835. 8vo. pp. 1238.

The diseases of the cutaneous surface have been long justly regarded as among the opprobria of medical science. Before the time of Willan and Bateman, this branch of pathology had obtained but little attention. The former sought to make it, what it never will be, a science almost *per se,* and the latter followed him in the attempt. By endless divisions and subdivisions where the laws of separation were seldom distinct, Willan defeated his object of rendering these affections better understood: he produced a complicated work, difficult to be understood, and sacrificed simplicity and clearness for technicality and diffuseness. Had the magnificent engravings with which his book is adorned been attainable on the terms they are in France, the chances of the extension of the knowledge of cutaneous diseases would have been materially increased, as the errors contained in it would have been more likely to be discovered. Nevertheless, the English writers are still the authorities to whom we must be content to refer; for M. Rayer has certainly not, in any essential degree, cleared up the difficulties with which Willan encumbered the subject.

The first edition of the present work was a very imperfect and crude production; the present, we may safely say, comprehends something of all which can fairly be brought under the designation of skin diseases, and a great deal more. The translator, Dr. Willis, has done ample justice to his original.

In his classification, M. Rayer has followed and copied Willan in all essential respects; he has, moreover, included a great number of diseases not originating in, and not necessarily connected with, those of the skin.

The general character of the work is that of an elaborate compilation, distinguished by extraordinary industry, and exhibiting an acquaintance with the writings of ancient and modern writers of all classes as regards dermatology. The introduction alone contains 150 references to other authors, and the volume itself has perhaps as many more as it contains pages of letter-press. The discretion and judgment with which many of these have been made do not appear quite so conspicuous; since they often refer to productions of very doubtful pretensions, and are more likely to puzzle the student than to enlighten him.

The first section contains *Inflammations having a single elementary form,* and comprises Erythema, Erysipelas, Rubeola, Scarlatina, Roseola, Urticaria, and several inflammations artificially produced.

"The common and generic anatomical character of these inflammations is the *red tint* in the parts of the skin affected; the red colour disappears on pressure, and returns immediately on its removal. The injection of the skin, which is slight in roseola and rubeola, and often very passing in urticaria, is more intense and permanent in erythema and erysipelas. Its principal seat is in the vascular network of the skin; but in erysipelas, urticaria, and even in rubeola and scarlatina, it will sometimes extend to the subcutaneous cellular tissue." (P. 97.)

This statement deserves the attention of the junior practitioner. The consideration of the actual seat of inflammation determines the general principles of treatment in all other cases; but, strange to say, in diseases of the skin, this circumstance has been overlooked by our best authors. The spreading of the cutaneous inflammation to the subjacent cellular structure, and the degree in which it takes place, may, under no very extraordinary circumstances, convert a case of erythema into simple erysipelas, which latter may speedily run into the gangrenous species of this disease; for, what is there in Willan's description of E. gangrenosum, but that of inflammation affecting alike deep-seated and superficial parts? As we shall have occasion to notice the nature and treatment of this interesting disease in another place, we pass it over here.

On the subject of Rubeola, Scarlatina, and Roseola, we do not find anything sufficiently important to notice; and there is nothing novel in the chapter on Urticaria.

*Bullæ, or Bullous Inflammation.* These are the affections designated, by Willan and Bateman, Pompholyx and Pemphigus. The undeviating external character of these diseases (probably we ought not to speak in the plural,) is a sudden appearance of vesicles on the extremities, on the first appearance not exceeding the size of a grain of wheat, increasing in a few hours to the size of a walnut; when the increasing distention of the cuticle leads to its rupture, and the fluid escapes, leaving an abraded cutis.

The structural changes, says M. Rayer, are those produced by burning or blistering; the causes obscure. Our author does not seem to be aware of the intimate connexion of this with the following section, Rupia: nevertheless, his remarks wear a practical character as regards both.

*Herpes.* "The anatomical structure of the vesicles and vesications of Herpes zoster may be studied during life, by opening them with the point of a pin or lancet. It will then be seen that, besides serum, the greater number of them contain a small piece of false membrane, which adheres very firmly to the vascular rete of the true skin below. The rete, of a vivid red colour, with small granulations formed by the papillæ scattered over its surface, occasionally presents minute points of a violet hue, especially under those vesicles that have been filled with bloody fluid. The quantity of serum effused is sometimes exceedingly small." (P. 255.)

The foregoing pathological remark, the correctness of which we do not doubt, was (as far as we know) first made by our author. We have often been surprised at the intense agony of feeling expressed by the sufferer under shingles, and surprised also at the rapid subsidence of the pain after a certain period. Is the false membrane a very superficial slough of the surface of the cutis? or is it simply an effusion of coagulated lymph mixed with the contents of the vesicle?

The commonly harmless termination of this affection, and its tendency to run its course despite of treatment, are well known; and we regret that there does not seem to be anything new offered by our author in a therapeutical point of view. Herpes zoster, or the "shingles," is the most gigantic of the family, and it is seldom in England found to degenerate in its course to anything like those forms mentioned by our author. It is seldom, in truth, here thought of any importance, although the details of many continental authors abound with descriptions which plainly connect it with formidable constitutional disease. The same, and perhaps a greater degree of violence of character will be found to prevail in cases of herpes in unhealthy localities, where the people are miserably fed and hardly worked. This feature of distinction may be traced so satisfactorily, and accounted for so clearly on physiological principles, that the most sceptical can hardly doubt. Thus, we see in the descriptions of Alibert and the author before us, as well as in many others, accounts of almost all diseases of the skin, so widely different from any which are observed in the British islands, that we doubt, and occasionally deny, their identity. We think we could satisfactorily illustrate this position by reference to the disease termed Pellagra, which we cannot help regarding as a very ordinary affection, aggravated into malignity by the peculiar circumstances of the people among whom it prevails: but our limits only permit us to allude to the circumstance.

*Scabies.* The late jubilee among the pupils of St. Louis, in consequence of the *discovery* of the itch insect, does not seem to have been contemplated by M. Rayer, any more than the necessity for M. Alibert's prize to the successful competitor. He appears to have exercised his usual powers of research on the subject among old authors, and the results do not, to our view, leave much to be proud of on the part of the alleged discoverer. This we know, moreover, that there are, or were some little time past, some six old women in St. Giles' Workhouse who would pick you out of the newly admitted children's skins as many as you desired.

*Acne.* By the term acne we have all been led in England to understand an *eruption of pimples* of a particular, or rather a peculiar kind. Willan places them in the order tubercula, because they present to the touch the feeling of a small hard granule or tubercle; but he subsequently intimates his conviction that they are the results of a part of a secreting organic structure becoming inflamed. Making the mistake of supposing a secreting follicle the excretory duct of a gland, he says the former becomes obstinate, and causes inflammation. The follicular apparatus of the upper parts of the body is, however, now better understood.

"Under the title of acne, then, I shall describe a chronic inflammation of the sebaceous follicles, common in youth and manhood, characterized by isolated acuminated pustules, most usually developed on the shoulders, sternal and scapular regions, the skin of which looks dense and unctuous, and more rarely on the face: these pustules are succeeded by livid or violet-coloured spots, by tuberculated indurations of the same or of a milky white hue, almost always intermingled with the accumulations of sebaceous matter with black points, vulgarly styled *worms*, and with follicular enlargements." (P. 464.)

"The mode in which the pustules of acne are formed, the different morbid features that almost invariably accompany the disease, the enlargement of the follicles on those regions of the skin where the acne appears, the immunity from this disease enjoyed by those districts unprovided with sebaceous follicles, such as the palms of the hands and soles of the feet, are so many circumstances that authorize us in assigning the follicles as the element of the skin which is particularly attacked in acne. This presumption, indeed, becomes a matter of certainty when the nascent and untouched pustules of the disease, (or those which are older,) after being laid open with the point of a lancet, are examined through a magnifying glass. Mr. Plumbe first satisfactorily demonstrated this anatomical fact; but he fell into an error when he maintained that the inflammation of the follicles was always excited and kept up by the accumulation of sebaceous matter within their cavities. The whole of the pustules of acne certainly do not appear as sebaceous concretions, or follicular elevations, at their outset: a certain number only commence in this way; the rest, from their origin, exhibit inflammatory characters (sanguineous injection, followed by the formation of pus), and blood or pus may frequently be extracted from their cavities, unmixed with indurated sebaceous matter." (P. 466.)

Now, when we consider that the follicles may be extremely minute, and that a correspondingly small portion of sebaceous matter may have obstructed the follicle, we can at the same time admit that a small portion of the same sebaceous matter may have become dissolved in the pus and blood which surrounded it. But the matter is not one of much practical importance.

*Sycosis.* This disease is hardly suspected to exist in England by those who have seen the frightful delineations of Willan and Alibert, (under the name of mentagra,) and others; it is nevertheless extremely common among all classes: it is acne of the chin—on a part covered by hair; it is constituted of inflamed follicles, and the practice of shaving keeps up that inflammation as long as it is continued.

"Sycosis is characterized by the successive evolution of a number of small pointed pustules, similar to those of rosacea, and scattered singly or clustered together over the chin, upper lip, submaxillary region, and lateral parts of the face. . . . Slight, partial, and passing pustular eruptions are usually observed to take place for several months, or even some years, upon the regions indicated, before a complete attack of sycosis occurs. In some rare cases, and almost always under the influence of appreciable causes, . . . the disease unexpectedly attacks the whole of the inferior maxillary region. The eruption is occasionally confined to the upper lip; at other times to one of the sides of the chin, to the lateral parts of the face, or to a part only of the submaxillary region; the disease in other instances attacks the whole of the regions indicated above, simultaneously or successively, and even extends to the roots of the hair in the nape of the neck, (*Sycosis capillitii.*) . . . On the second or third day of their formation, the tops of these elevations grow white, and are filled with a pale yellowish pus; they subsequently increase a little, but it is seldom that they surpass a millet-seed in size. Almost all of them seem traversed by a hair; they do not discharge like those of impetigo. Between the fifth and seventh day each pustule bursts spontaneously, its sides shrink, and a slight oozing takes place, which gives rise to a brownish crust that scarcely adheres to the skin, and is confounded at its edges with the epidermic furfuræ which are thrown off by the inflamed integument in the vicinity of the pustule." (P. 481.)

Even this description scarcely applies to anything which is seen in the better classes of society in this country, and what follows, under circumstances favorable to the extension, is, we are thankful to say, entirely unknown among us.

"When the pustules are grouped or clustered in considerable numbers, the inflammation extends to the cellular membrane immediately under the inflamed portion of corion, and gives rise to a true phlegmonous swelling. The chin, the sub-maxillary regions, and the upper lip, then present small, hard, painful red *tumours*, covered with pustules or incrustations of considerable thickness, and a mixed yellow and greenish brown hue. . . . When the pustules are thrown out repeatedly on the same places, the inflammation extending from the corion to the subcutaneous cellular membrane, there occasions indurations which before long present the appearance of voluminous tubercles. . . When the eruptions have been copious and severe, and have succeeded each other rapidly, these tubercles increase in number, and spread over the whole extent of the chin. . . . It is then particularly that the confused mixture of pustules, tubercles, and incrustations, give a disgusting character to the appearance of sycosis. Arrived at this stage, sycosis is always an obstinate disease, the cure of which is never obtained but with great difficulty. The skin occasionally becomes very much altered, and swells to such a pitch as to appear covered with moist and vegetating tumours." (P. 482.)

*Impetigo.* This is, perhaps, among the most common of skin diseases in this country. Among the better classes of society, if we except lepra, it is more frequently seen than any other. The author observes that it is most frequently seen on the face, and next in order on the neck, trunk, and extremities. The reverse of this is certainly the case in England; as the extremities, particularly the lower, are its chief seat. M. Rayer adopts the divisions and terms of Willan: sparsa, figurata, &c. He has mixed the crusta lactea, or porrigo larvalis, under one general head, with the other forms of this disease; and it is more than probable that his judgment is correct in this respect. This disease is certainly not allied by any essential characters to porrigo; but it is equally true that it is widely different from the impetigo of any other period of life.

*Porrigo.* Of the varieties of the affections usually classed under this term, we find in our author scarcely more than the names. With the exception before alluded to, he comprehends all under the term *favus.* Here again the apprehension that we are studying the diseases of a dirtier class of paupers than any in England presses on us.

"The word *favus*," says M. Rayer, "is used to delineate a chronic inflammatory affection of the skin, essentially contagious in its nature, and principally characterized by the appearance of its scabs, which are of a clear yellow colour, very dry, strongly adherent to the skin, circular and cupped, and either isolated or agglomerated into continuous masses with raised and inverted edges; the surface of which presents numerous characteristic depressions." (P. 510.)

"The smell of the scabs and incrustations of favus is singularly like that of the urine of the cat. When they are softened with emollient cataplasms, the smell changes and becomes faint and sickly, and something similar to that of bones which have been boiled with their ligaments." (P. 512.)

"Pediculi are usually found in vast numbers among the crusts of favus; and children seem to taste a sort of ecstasy in tearing their scalp with their nails. The blood mixed with the discharge poured out in these and similar cases, by drying, forms incrustations of a colour different from that presented by the ordinary crusts of favus." (P. 513.)

If there are such cases in England often seen, we are very much mistaken; the progress of formation of such accumulated masses of disgusting morbid secretions would not be permitted by the poorest parent of a family, and it would be a libel, we trust, on our lowest poor-house to say that it contained an inhabitant presenting such a loathsome state of artificial disease.

The various theories which have been formed as to the seat of this disease are next discussed, as also its pathology; in neither of which discussions we find anything novel. At length we arrive at the "depilatory plan" of treating it, which is said to be, in old standing cases, "as indispensable as is the removal of the nail in certain varieties of onychia."

The well-known effect of the old pitch cap had long proved to us the correctness of this assertion, but how horrible was the suffering it inflicted; applied to the poor child's head, and attached alike to the sound hair of the healthy parts and that of the diseased, and torn with violence away, uprooting at one fell swoop all within its merciless entanglements, despite the screams and agony of the sufferer! To avoid this process, a pair of forceps was constructed by Mr. Plumbe; but our author states, in speaking of them, "that this procedure, which lasts infinitely longer than the other, is in itself excessively painful when the hairs still adhere to their bulbs." (P. 523.) We believe Mr. Plumbe cautiously avoided the use of his forceps when any attachment of the hair sufficient to give pain remained.

Of all the depilatory methods proposed, says M. Rayer, "that of the Messrs. Mahon is unquestionably the best." But the Messrs. Mahon, it seems, sensible of its value, persist in keeping the said depilatory method a secret; and the physicians and surgeons of the Hospital of St. Louis permit two nostrummongers to traverse the wards of their hospitals, not merely uncontrolled, but, it would appear from our author, venerated and respected. This is certainly a state of things to be much deprecated, for the honour and dignity of our profession. Surely, if the method is really so valuable as is stated by the French physicians, (and not only our author but Alibert and Biett declare that no other means of cure can compete with it,) the secret ought to be purchased by government, and made known for the good of the public.

The translator, we observe, in a note, states that the chief secret of MM. Mahon's success consists of a long and persevering attention to cleanliness. That it is a very essential point there is no doubt, but there is evidently more than this. There is a secret depilatory powder and a secret depilatory ointment employed, and the imitation or analysis of them appears not likely to be accomplished by any of the French savans, seeing that the said secrets have been kept so long, and continue to be so well paid for.

*Ecthyma.* We cannot say that M. Rayer has been so successful in the elucidation of the etiology or pathology of ecthyma as Willan and Bateman were before him, in their division of the disease into four species, the common, the lurid, the infantile, and cachectic. Thus, they observe that E. vulgare "commonly supervenes on a state of languor of some continuance, with loss of appetite, irregularity of the alvine evacuations, and pains in the stomach and limbs." Then, "the E. infantile occurs in weakly infants, when insufficient nourishment is afforded them. The E. luridum again is remarkable for the dark red colour of the bases of its pustules, and "is most frequently seen in persons of advanced age, who have injured their constitutions by hard labour or intemperance; and is most severe in the winter season." Dr. A. T. Thomson, in his late edition of Bateman, says that "a symptomatic ecthyma, which bears a considerable analogy to E. luridum, sometimes occurs during the cachectic state which follows the measles, as well as the other debilitating exanthemata." A consideration of the several varieties of this disease suggests the question, whether they might not be all more satisfactorily included under some more general term, implying the sense of "cachectic disease of the skin."

No less than thirty pages are occupied by an account of diseases which can hardly be considered as properly those of the skin; such as the stye, the carbuncle, and malignant and gangrenous anthracion. The article on the latter, however, is well worthy of perusal.

In the account of *Lichen* and *Strophulus,* the two first genera in M. Rayer's order *Papulæ,* there is little to be found in addition to what is supplied by English authors; and, indeed, this part of the work is more remarkable for a free appropriation of their labours than any improvement of their views. Their faults as well as their excellencies have been freely copied, and the most trifling papular eruptions of infants assume, in M. Rayer's work, all the importance they obtained in those of Willan and Bateman.

This class of eruptions is seldom the result of any cause in operation in the system more powerful than a little temporary irritation from disordered stomach or teething combined with repletion. Perhaps an attempt to arrange the etiology of cutaneous diseases would properly commence with these, as the first links of a chain, the last or most extreme parts of which would be constituted of ecthyma, rupia, and such others as originate in scorbutic and impoverished conditions of the system. The results of the methods of treatment, too, which such a view would naturally suggest, are, although equally simple, equally widely different from each other. None of these exhibit obstinacy of character; they seldom defy the efforts of the practitioner for any length of time, or give occasion to much suffering to the patient.

Of *prurigo,* certainly one of the most intractable and intolerable of diseases, the author has given a clear and accurate account.

Simple or sulphureous baths, those of soap and alkalies, of sea water, of gelatine and sulphur combined, ointments of hellebore and hydrochlorate of ammonia, bleeding, diluents, liquids, low diet, and purgatives, constitute the chief sources of dependence in the treatment of the disease; where it is general, the author observes, that external applications have appeared to him so advantageous that he recommends, except in a few cases, the treatment to be confined entirely to them.

*Squama. Scale.* "It is the distinguishing character of the squamous inflammations to appear as red elevations, spots, or blotches, upon which *squamæ,* in other words, laminæ of the cuticle altered in various degrees, are formed, thrown off, and incessantly renewed. The number of squamous inflammations reckoned is six: lepra, psoriasis, pityriasis, pellagra, acrodynia, and scaly syphilis. . . . I have . . separated from the group of pityriasis two varieties of cutaneous affections, described by Willan under the heads Pityriasis versicolor and Pityriasis nigra. These I denominate Chloasma and Melema, as I hold that they belong essentially to the class of pigmentary affections. Mr. Plumbe and Dr. Duffin have proposed to unite the description of lepra to that of psoriasis. To place the distinguishing characters of these two diseases in greater relief, I have continued to describe them separately, although I still acknowledge the striking analogy that exists between their various symptoms. Inflammation of the reticular tissue and papillæ is the first and main feature of squamous affections." (P. 614.)

M. Rayer, while writing the above, appears to have forgotten that the pigmentary membrane has been denied at least, if not proved to have no existence, by the highest authorities in his own country. His transposition of P. versicolor and P. nigra, on the grounds alleged, will hardly therefore be considered justifiable; and we are decidedly of opinion that the external characteristics of the squamous eruptions, one and all, may be more satisfactorily accounted for than by attributing them to the derangement of structures of doubtful existence.

Attention to the general health, combined with tonics, among which arsenic, as known of old amongst us, constitutes the most powerful remedies which M. Rayer is aware of in these diseases.

The *tubercular inflammations* are characterized at their height by small, solid, circumscribed, indurated, and enduring tumours, which, lasting for months or years, end by becoming ulcerated. They are six in number: Lupus, Cutaneous Scrofula, Cancer, Greek Elephantiasis, Tubercular Syphilis, and artificially excited Tubercles, the group differing essentially from that of the same designation by Willan and Bateman. These different diseases are each treated of at some length. Their description and history are accurately given; but our readers will have anticipated that they, for the most part, are as intractable in the hands of our French brethren as in our own. We do not discover among the remedial measures any thing to which we were previously strangers. The reader will, notwithstanding, find the whole well worthy of his attention.

We pass over a long account of several important diseases which cannot properly be classed among cutaneous affections, such as, Anæmia, Chlorosis, Scorbutus, Purpura,| Leucopathy, artificial staining of the Skin by Nitrate of Silver, &c.

Of this latter singular affection our author has given us the results of a post-mortem examination of a man twenty-eight years of age, who had taken the medicine for thirteen months for the cure of epilepsy, consequent on disease of the brain.

"All the external integument was of a grey slate colour of moderate intensity. This hue, which was nearly the same in all parts of the skin, did not prevent the vascular colour of the cheeks from being distinguished. The edges of the lips, their internal surface, the inside of the cheeks, and both sides of the tongue, presented an exactly similar hue; the internal surface of the whole alimentary canal was of the same colour as the skin, and the upper opening of the gastro-pulmonary membrane. In the stomach this tint was extremely deep; it was not mixed with any violet-coloured marblings, depending on vascular patches or striæ; it was uniform over the whole extent of the viscera. In both the small and great intestines it was a little clearer, but still very appreciable: it was uniform as in the stomach, and slight traces only of vascular ramifications were discovered in the whole extent of the alimentary canal." (P. 963.)

The impression seems to have been entertained by different authors that this discoloration does in time diminish in intensity; if so, this change must take place very slowly. We recollect hearing it stated by the late respected Dr. Babington, than whom no man could be better qualified to give evidence on the subject, that, in the course of his long professional life, he had never known that stain disappear or change. It behoves the practitioner therefore carefully to deliberate before he adopts such a medicine as this.

There is no novelty whatever in the account of Ichthyosis. Horny Productions, Cicatrices, Gangrene, Carbuncle, Onychia, Grease, Scabies of Animals, &c. occupy the remainder of the pages; and these we must pass over.

We take leave of our author with the declaration that his work is a monument of the most extraordinary industry. We have no hesitation in adding that, although far from faultless, it is the best book we possess, in any language, on the subject; and that, should any of our readers desire to sail over the unbounded sea of letter-press formed of the history and pathology of the diseases of the cutaneous surface, M. Rayer should be his pilot.

Nevertheless, a great book is a great evil; and this axiom applies forcibly to the work of M. Rayer. He has, moreover, in our opinion, committed a great error, treading in the steps of several of his predecessors, in so multiplying, dividing, and subdividing simple affections, differing only in stage or degree, into species and varieties, (giving each a separate designation,) which have no foundation in reality. It is evident that this practice is destitute of utility. That it is injurious to the advancement of knowledge of these diseases is manifest, because it confuses and discoura es the student, let his zeal be what it may in its pursuit. A sensible and well-conducted attempt to bring to this subject the simpler means of illustration and description employed in other branches of pathology is still wanting; and, if the subject were confined to the history, pathology, and treatment of the diseases of the skin as they appear in the British islands, a work of far more utility for practitioners in this country would be the result than that furnished by M. Rayer.

We cannot conclude this article without special and particular notice of the Atlas of Illustrations, which forms so important a part of M. Rayer's work.

That which strikes us most in glancing at M. Rayer's atlas is the immense number of representations which it contains of the various acute and chronic diseases properly belonging to the skin, besides a great many of other diseases which dermatologists in general are not disposed to consider as belonging to the pathology of this tissue, such as cancer, melanosis, horny productions, chancres, &c. However, this addition to the atlas of cutaneous diseases, properly so called, must, as we have already stated, be regarded as a proof of the industry and zeal of the author, and as evincing a desire on his part to render the subject of which he treats as complete as possible. Considered in this respect, but more especially in reference to the number of illustrations of the general species and varieties of each order which it contains, this atlas far surpasses any that has yet appeared.

With regard to the illustrations furnished by the individual plates of the particular diseases belonging to each order, most of the subjects from which they have been taken appear to have been well chosen; many of them have been faithfully represented; others less so, either from a defect on the part of the draftsman or the colourist. There is no tissue, the faithful representation of whose diseases depends so much on the aid of colour as the skin; the species and varieties being often founded on the presence or absence of colour; and different shades of the same colour, particularly red, serving as a distinguishing feature in several of the stages of the exanthemata, by marking the character and degree of the inflammation by which they are accompanied. The syphilitic nature of many cutaneous eruptions is determined by the peculiarity of their colour (*couleur cuivrée*); and none so conspicuously as that which follows the internal exhibition of the nitrate of silver. The technical term of *dirty* or *muddy* colouring applies to several of the plates in this atlas; but, as a whole, it presents a good specimen of the French school; and, although most of the legs, faces, &c. are curtailed of their "fair proportions" and unadorned, their infirmities are more true to nature than those of the beauties which we all admire in the grand work of M. Alibert. Representations of cutaneous diseases on a small scale, that is to say, in *bits* or *squares*, or what is worse, on a *diminished* scale, possess little value, or mislead the student; and this our author appears to have avoided, as far as the size of the atlas and the number of figures it contains, would permit. The plates of Willan and Bateman, and of Alibert, strike us at first sight as being more natural than those of Rayer; but, had those of this author been done on the same scale, with that sort of frame-work which a part of the dress always forms to a picture of even a portion of the human figure, they would have gained much, if not as specimens of art, as more impressive, because more accurate representations of nature.

It is in consequence of a total want of these pictorial accessories that Dr. A. T. Thomson's Atlas, although better coloured than that of Rayer, conveys but an imperfect and indefinite idea of cutaneous diseases to the mind of the student. On the whole, Rayer's atlas may conscientiously be said to contain the most complete series of illustrations of cutaneous diseases hitherto published, and is, besides, not only cheaper than any other, but well worth the sum for which it is offered to the profession.

---

